# Equity and efficiency of maternal and child health resources allocation in Hunan Province, China

**DOI:** 10.1186/s12913-020-05185-7

**Published:** 2020-04-15

**Authors:** Minyuan Huang, Dan Luo, Zhanghua Wang, Yongmei Cao, Hua Wang, Fengying Bi, Yunxiang Huang, Luxi Yi

**Affiliations:** 1 Department of Social Medicine and Health Management, Xiangya School of Public Health, Central South University, Changsha, 410078 People’s Republic of China; 2 Health Commission of Hunan Province, Changsha, 410008 People’s Republic of China; 3 Hunan Provincial Maternal and Child Health Care Hospital, Changsha, 410008 People’s Republic of China

**Keywords:** Maternal and child health resources allocation, Equity, Efficiency, Gini coefficient, Data envelopment analysis, Hunan Province, China

## Abstract

**Background:**

A reasonable allocation of health resources is often characterized by equity and high efficiency. This study aims to evaluate the equity and efficiency of maternal and child health (MCH) resources allocation in Hunan Province, China.

**Methods:**

Data related to MCH resources and services was obtained from the Hunan maternal and child health information reporting and management system. The Gini coefficient and data envelopment analysis (DEA) were employed to evaluate the equity and efficiency of MCH resources allocation, respectively.

**Results:**

The MCH resources allocation in terms of demographic dimension were in a preferred equity status with the Gini values all less than 0.3, and the Gini values for each MCH resources’ allocation in terms of the geographical dimension ranged from 0.1298 to 0.4256, with the highest values in the number of midwives and medical equipment (≥ CNY 10,000), which exceeds 0.4, indicating an alert of inequity. More than 40% regions in Hunan were found to be relatively inefficient with decreased return to scale in the allocation of MCH resources, indicating those inefficient regions were using more inputs than needed to obtain the current output levels.

**Conclusions:**

The equity of MCH resources by population size is superior by geographic area and the disproportionate distribution of the number of medical equipment (≥ CNY 10,000) and midwives between different regions was the main source of inequity. Policy-makers need to consider the geographical accessibility of health resources among different regions to ensure people in different regions could get access to available health services. More than 40% of regions in Hunan were found to be inefficient, with using more health resources than needed to produce the current amount of health services. Further investigations on factors affecting the efficiency of MCH resources allocation is still needed to guide regional health plans-making and resource allocation.

## Background

The maternal and child health (MCH) care is the key component of almost every health system in worldwide. It is also an essential part of the 2030 Sustainable Development Goals (SDGs) proposed by World Health Organization (WHO) in 2015, which includes targets to “reduce the global maternal mortality ratio and deaths of newborns and children under 5 years of age” [1]. In China, after the annunciation of the universal “two-child” policy (a policy allowing all couples to have two children) in October 2015, the need and demand for MCH services has been increasing accordingly [2]. The Chinese government has been attaching great importance to MCH and issued “Healthy China 2030”, a national health plan, in 2016, with a primary goal of improving the health status of mothers and children [3].

There has been a remarkable improvement in MCH in China over the last decades. The maternal deaths declined from 111.0 per 100,000 live births in 1990, to 21.8 per 100,000 live births in 2015, and the mortality rate for children under 5 years of age also went down from 54.1 per 1000 live births to about 12.5 per 1000 live births during the same period [4]. However, the progress has been uneven and inequitable at different regions, with higher maternal and child mortality rates in low- and middle-income areas [4]. While there have been studies suggesting that these geographic disparities may result in part from the differences in the degree of equity and efficiency in allocating MCH resources among different regions [5, 6].

Equity in the health resources allocation means that the way health resources distributed among the health care departments or regions can reflect the degree of health fairness [7]. The efficiency refers to the relationship between health inputs and health outcomes. Efficient health resources allocation means that how to produce the greatest health outcomes for a given set of health resources or how they generate the ideal health outcomes using minimal resources [8]. Ensuring equity and efficiency in the allocation of health resources is one of the basic conditions to achieve the equitable, effective and quality health services for mothers and children.

The question about how health resources should be allocated, so that they are distributed in both the most equitable and most efficient way should be an important concern in MCH system. Studies to date, however, have mainly focused on the equity and efficiency of health resources allocation in primary health care [9, 10], overall health care [11, 12], or specific health-care institutions such as public hospitals [13] and county hospitals [6]. Although some studies focus on the MCH resources allocation in China, most studies was conducted in lower- or higher- income regions, with little information available in the middle-income regions [14–17]. Whereas, assessing the equity and efficiency in MCH resources allocation in different regions is necessary because the problems of determining the appropriate allocation of MCH resources between regions at different levels of economic development may be different.

Hunan Province (24°39′-30°08′ N, 108°47′-114°15′E), located in the central south of China, covers an area of around 212, 204 km^2^ and has a population of approximately 74 million as of 2017. The estimate of the gross domestic product (GDP) per capita for Hunan in the same year is about 49,558 yuan, which is regarded as a typical middle-economic-level area according to the rankings of provincial GDP per capita in China [18]. It includes 14 regions with Changsha as its capital. In 2017, there are 137 MCH hospitals (MCHHs) at different levels in Hunan, including 1 provincial-, 14 municipal- and 122 county-level MCHHs. The maternal and under-five mortality rate in Hunan in 2017 were 12.7 per 100,000 live births and 4.86 per 1000 live births respectively, which are 2.24 and 1.84 two times higher, respectively, than the rates in Beijing, a developed eastern region in China [19, 20]. There is an urgent need to evaluate the equity and efficiency of MCH resources allocation in Hunan to provide the scientific basis of regional health planning to narrow the regional disparities in MCH outcomes.

The evaluation of equity and efficiency in the allocation of MCH resources is an important part that can help health planners and decision-makers identify bottlenecks and take appropriate actions to further optimize the allocation of limited health resources. This study aimed to assess the equity and efficiency of the MCH resources allocation in Hunan, China, to explore potential measures for optimizing the MCH resources allocation.

## Methods

### Data sources and statistical analysis

The geographic area data was sourced from the 14 municipal government websites in Hunan. Data related to MCH resources and services as well as the population numbers (2017) was obtained from the Hunan maternal and child health information reporting and management system. This system is a network data reporting system for Hunan maternal and child health surveillance which was established in 2004. The data in this system is originally collected by the staff responsible for maternal and child care in primary health care institutions and then upload it to the system, which is further audited and managed by Health Commission of Hunan Province. The administrative permission from Health Commission of Hunan Province is required to access and use the data in this system. We had received permission to use these data before data analysis in this study.

Microsoft Excel 2016 was used to calculate the Gini coefficient, and DEAP (V2.1) was employed to conduct the data envelopment analysis (DEA) [12].

#### Gini coefficient

The Gini coefficient is regarded as a superior tool for evaluating the equity of health resources allocation in demographic and geographical dimensions [21]. It is derived from the Lorenz curve, indicating the ratio of the area between Lorenz Curve and the 45° line to the whole area below the 45° line. The formula to calculate the Gini coefficient is presented as follows [9]:

$$ G=1-\sum \limits_{i=0}^{k-1}\left({Y}_{i+1}+{Y}_i\right)\left({X}_{i+1}-{X}_i\right) $$

*Y*_*i*_ is the cumulative percentage of MCH resources (the eight indicators to assess the equity) in the *ith* region; *X*_*i*_ is the cumulative percentage of population or geography in the *ith* region; *k* is the total number of regions; *G* is the value of the Gini coefficient.

The value of Gini ranges from 0 to 1, with higher score indicating greater inequity. Generally, a value of 0 means the absolute equitable allocation of resources; < 0.3 represents preferred equity status; 0.3–0.4 is the normal condition; > 0.4 refers to an alert of inequity (relative inequity); and > 0.6 indicates a severe inequity [10, 21].

#### DEA model

DEA is a non-parametric method that can evaluate the relative efficiency of decision-making units (DMUs), incorporating the multiple input and output variables [22]. The Charnes, Cooper, and Rhodes model (CCR model) and the Banker, Charnes, and Cooper model (BCC model) are considered as the two most superior tools to measure the relative efficiency [12]. The CCR model assumes that production is constant return to scale and can calculate the overall efficiency score, while the BCC model assumes that production is variable return to scale which can further decompose the overall efficiency score of the CCR model into pure technical and scale efficiencies.

In addition, the projection value of the input and output indicators of the inefficient DMUs can be also estimated by BCC model. By measuring the distance from the inputs and outputs of inefficient DMUs to their efficient peers, we could calculate the over-supplied inputs and expected outputs for the inefficient DMUs, which can provide the quantitative basis for improving the efficiency of MCH allocation of each DMU. Thus, we preferred to select the BCC model in this study to evaluate the efficiency of MCH resources allocation [9, 23].

DEA models include input- and output-oriented models. An input-oriented model reflects the degree to which a DMU can reduce its input without changing its outputs, while an output-oriented model indicates the degree to which a DMU can expand its output without changing its inputs [24]. In this study, the outputs are associated with the quantity of maternal and child health services. With the implementation of the “two-child policy”, the demand for maternal and child health services has been increasing [2]. Thus, we chose output-oriented model.

In this study, every region of Hunan Province was assumed as a DMU, with 14 DMUs in total. We used the algebraic sum of the inputs/outputs of all the MCHHs to estimate the efficiency at a region level, except for the system management rate for children under 3 years old. An averaged system management rate was calculated for each of the regions. The number of MCHHs in 14 regions of Hunan is shown in Table 1. By taking Changsha as an example, Changsha has 11 MCHHs, the number of beds in Changsha region is the sum of the beds of 11 MCHHs, totaling 2409 beds in Changsha.

**Table 1:** The number of MCHHs in 14 regions of Hunan in 2017

Regions	Provincial - level	Municipal - level	County-level	Total number
Changsha	1	1	9	11
Xiangtan	0	1	5	6
Zhuzhou	0	1	9	10
Yueyang	0	1	9	10
Changde	0	1	8	9
Chenzhou	0	1	11	12
Hengyang	0	1	12	13
Yiyang	0	1	6	7
Loudi	0	1	5	6
Zhangjiajie	0	1	4	5
Yongzhou	0	1	11	12
Huaihua	0	1	13	14
Shaoyang	0	1	12	13
Xiangxi	0	1	8	9
Total number	1	14	122	137

The efficiency status of 14 DMUs was estimated by the values of overall efficiency, technical efficiency and scale efficiency. Overall efficiency means that under the same circumstances, the actual output levels of a DMU is similar to the ratio of maximum output [11]. Technical efficiency is the ratio of the minimum input to the actual input levels of a DMU for a given set of outputs, keeping the input proportion constant [11]. Scale efficiency represents the constant evaluation of the scale return, the multiple output increase as equivalent to the increase in multiple inputs [11]. The following is a formula showing the relationship between the three [11]:

$$ \mathrm{Overall}\ \mathrm{efficiency}=\mathrm{Technical}\ \mathrm{Efficiency}\ast \mathrm{Scale}\ \mathrm{Efficiency} $$

The value of overall efficiency, technical efficiency and scale efficiency all range from 0 to 1. When overall efficiency is 1, the DMU is relatively efficient; when technical efficiency is 1, but scale efficiency and overall efficiency are less than 1, the DMU is weakly efficient; when the overall efficiency ranges from 0 to 1, the DMU is inefficient [12].

### Indicators

A comprehensive literature review was conducted in the process of indicators selection [11–13, 25]. Additionally, experts including health administrators working in Health Commission of Hunan Province, the MCH doctors working in Hunan Provincial Maternal and Child Health Care Hospital were invited to review the rational of indicators selection by telephone consultations.

#### Equity

A total of 8 indicators were selected in this study to evaluate the equity in MCH resources allocation. Human resources and capital are regarded as the important input/output variables in the delivery of health services [9]. The number of doctors, nurses, midwives, and administrative staff members represents human resources, and the MCHHs, beds, operating areas, medical equipment with a value of ≥ CNY 10,000 (above $1420) represents the capital. Medical equipment (≥ CNY 10,000) is also an important indicator reflecting the hardware investments. The definition of equity indicators is shown in Table 2.

**Table 2:** Definition of equity indicators

Variable	Definition
Number of MCHHs	At the end of the year, the sum number of maternal and child health hospitals.
Number of beds	The practical beds in MCHHs (also referred to the number of actual beds).
Operating areas	At the end of the year, all housing areas of MCHHs except for the residences of employees, includes the areas for medical services, public health services, medical education and scientific research, logistical support, administrative management and hospital life facilities.
Number of medical equipment with a value of ≥ CNY 10,000	At the end of the year, the amount of medical equipment with a unit price exceeding CNY 10,000 in MCHHs.
Number of doctors	At the end of the year, the sum of doctors and assistant doctors in MCHHs who have obtained registration certificates and are actually engaged in disease prevention and control, medical treatment, or maternal and child health care.
Number of nurses	At the end of the year, the sum of nurses who have obtained the certificate of registered nurses and are actually engaged in nursing work in MCHHs.
Number of midwives	At the end of the year, the sum of health personnel who have received professional education in midwifery, mastered normal delivery, neonatal treatment, dystocia treatment, and basic theoretical knowledge and skills in maternal and child health.
Number of administrative staff members	At the end of the year, the sum of the staff members who are responsible for leading or managing tasks in medical and health institutions, includes those who are engaged in the management of medical tasks, public health, medical scientific research and teaching, and those who are engaged in the administrative work of party and government, personnel, finance, statistics, information, security, and so on.

#### Efficiency

In terms of efficiency assessment, 4 indicators including the number of health personnel, beds, operating areas, and the number of medical equipment (≥ CNY 10,000) were selected as inputs. The first indicator represents human resources, and other three represent capital. Another 3 output indicators are the number of live births, outpatients and emergency visits, system management rate for children under 3 years old. The definition of efficiency indicators is shown in Table 3.

**Table 3:** Definition of efficiency indicators

Category	Variable	Definition
**Input**	Number of health personnel	At the end of the year, the sum of registered doctors, registered assistant doctors, registered nurses, pharmacists, laboratory and imaging technicians, health supervisors and probationers in MCHHs.
	Number of beds	The practical beds in medical and health institutions (also referred to as the number of actual beds).
	Number of medical equipment with a value of ≥ CNY 10,000	At the end of the year, the amount of medical equipment with a unit price exceeding CNY 10,000 in MCHHs.
	Operating areas	At the end of the year, all housing areas of MCHHs except for the residences of employees. This includes areas for medical services, public health services, medical education and scientific research, logistical support, administrative management and hospital life facilities.
**Output**	Number of live births	The number of newborns of more than 28 weeks’ gestation age with one of the four vital signs after delivery: breathing, heart beats, pulsation of the umbilical cord and definite movements of voluntary muscles.
	Number of outpatients and emergency visits	The number of patients having outpatient and emergency diagnostic services.
	System management rate for children under 3 years old	The proportion of children under 3 years of age receiving regular physical examinations within the stipulated time.

## Results

### General information on MCH resources

There were 137 MCHHs in Hunan Province in 2017, which includes an operating area of 1,169,480 square meters and a total of 13,141 beds. The total number of MCH workers were 11, 626, including 8224 doctors, 1087 nurses, 1164 midwives and 1151 administrative personnel. Additionally, the number of medical equipment (≥ CNY 10,000) was 18,441 (See Additional file 1 for further detail).

### The equity of MCH resources allocation

The MCH resources allocation in terms of demographic dimension were in a preferred equity status with the Gini values all less than 0.3. The most equitable resource allocation was the number of MCHHs with the lowest Gini value (0.1325) and the most inequitable was midwives with the highest Gini value (0.2950).

The Gini values for each MCH resources’ allocation in terms of the geographical dimension ranged from 0.1298 (highest equity) in the number of MCHHs to 0.4256 (lowest equity) in the number midwives. The allocation equity of medical beds, operating areas, doctors, nurses and administrative personnel allocation was in a normal condition, with Gini values fluctuating between 0.3 and 0.4. While the Gini value for midwives and medical equipment (≥ CNY 10,000) was 0.4256 and 0.4077, respectively, which exceeds 0.4, indicating an alert of inequity.

In general, the MCH resources allocation seem to be more equitable in the demographic dimension than that in the geographical dimension. Except for the MCHHs, the Gini values for the allocation of doctors, nurses, midwives, administrative personnel, operating areas and medical equipment (≥ CNY 10,000) per thousand persons were generally lower than that in terms of per square kilometers. The Gini values for MCH resources allocation based on demographic and geographical dimensions are shown in Table 4.

**Table 4:** The Gini values for MCH resources allocation in Hunan in 2017

The Gini coefficient	MCHHs	Number of beds	Operating area	Medical equipment *	Number of doctors	Number of nurses	Midwives	Administrative personnel
Demographic	0.1325	0.2107	0.2222	0.2790	0.1923	0.2253	0.2950	0.2250
Geographical	0.1298	0.3673	0.3358	0.4077	0.3395	0.3767	0.4256	0.3498

^*^ Number of medical equipment (≥ CNY 10,000)

### The efficiency of MCH resources allocation

Table 5 presents the efficiency values in MCH health resources allocation of the 14 regions in Hunan Province. The average scores of overall efficiency, technical efficiency, and scale efficiency in the 14 regions were 0.900, 0.991 and 0.907, respectively. Among the 14 regions, 4 regions (28.6%), including Changsha, Hengyang, Zhangjiajie and Xiangxi, had overall efficiency scores of 1, indicating that the MCH resources allocation in these regions is relatively efficient. Another 4 regions (28.6%), including Zhuzhou, Xiangtan, Shaoyang and Chenzhou, had technical efficiency scores of 1, but overall and scale efficiency scores of less than 1, indicating the MCH resources allocation in these regions is weakly efficient when compared with efficient regions. Additionally, the other 6 regions (42.8%), including Yueyang, Changde, Yiyang, Yongzhou, Huaihua and Loudi, had overall efficiency, technical efficiency and scale efficiency scores of less than 1, suggesting that the MCH resources allocation in these regions was inefficient. Among the 10 relatively scale-inefficient regions, all of them had decreasing return to scale (DRS), indicating that these regions had scales that were too large and needed to cut down their operations to achieve constant returns to scale.

**Table 5:** Efficiency values and returns-to-scale characteristics of the 14 regions in Hunan in 2017

DMUs	Overall efficiency	Technical efficiency	Scale efficiency	Type of scale inefficiency	Relatively efficiency status
Changsha	1.000	1.000	1.000	–	Efficient
Hengyang	1.000	1.000	1.000	–	Efficient
Zhangjiajie	1.000	1.000	1.000	–	Efficient
Xiangxi	1.000	1.000	1.000	–	Efficient
Zhuzhou	0.962	1.000	0.962	Drs	Weakly efficient
Xiangtan	0.912	1.000	0.912	Drs	Weakly efficient
Shaoyang	0.960	1.000	0.960	Drs	Weakly efficient
Chenzhou	0.903	1.000	0.903	Drs	Weakly efficient
Changde	0.756	0.984	0.769	Drs	Inefficient
Huaihua	0.782	0.947	0.826	Drs	Inefficient
Loudi	0.784	0.979	0.801	Drs	Inefficient
Yueyang	0.793	0.983	0.807	Drs	Inefficient
Yongzhou	0.876	0.990	0.884	Drs	Inefficient
Yiyang	0.878	0.996	0.881	Drs	Inefficient
Mean	0.900	0.991	0.907	/	/

*Drs* Decreasing return to scale

In the BCC model, we also calculated the projection value of the input and output indicators of the inefficient regions (Table 6). The inefficient regions should either decrease their inputs or increase their outputs to improve the efficiency of MCH resources allocation. The result from input-output projection analysis of Changde, for example, indicates that, in order to achieving a relatively optimal resources allocation, Changde could reduce the average number of health personnel by 104.3, the average number of beds by 45.91 and the average number of medical equipment (≥ CNY 10,000) by 251.7, when keeping its present output levels unchanged. Alternatively, Changde needs to increase the average number of live births by 281.2, the outpatient and emergency visits by 128,874.1 and the system management rate for children under 3 years old by an average of 1.48% at the current input levels.

**Table 6:** Input-output projection analyses in 6 inefficient regions

Regions		Y_**1**_	Y_**2**_	Y_**3**_	X_**1**_	X_**2**_	X_**3**_	X_**4**_
Changde	Actual value	17,456.00	939,513.00	91.57	1689.00	1042.00	85,664.52	1560.00
	Projection value	17,737.21	1,068,387.10	93.05	1584.70	996.09	85,664.52	1308.30
	Insufficient or redundancy value	281.21	128,874.1	1.48	−104.3	−45.91	0.00	−251.7
	Insufficient or redundant rate (%)	1.61	13.72	1.62	−6.18	−4.41	0.00	−16.13
Huaihua	Actual value	8138.00	768,498.00	88.35	1534.00	806.00	122,455.80	1149.00
	Projection value	12,589.82	811,314.76	93.27	1191.84	760.21	67,589.84	1023.26
	Insufficient or redundancy value	4451.82	42,816.76	4.92	− 342.16	−45.79	−54,865.96	− 125.74
	Insufficient or redundant rate (%)	54.70	5.57	5.57	−22.31	−5.68	−44.81	−10.94
Loudi	Actual value	16,147.00	742,479.00	89.70	1693.00	1101.00	69,414.12	1415.00
	Projection value	16,490.74	758,285.04	91.61	1413.68	856.35	69,414.12	1049.72
	Insufficient or redundancy value	343.74	15,806.04	1.91	−279.32	− 244.65	0.00	− 365.28
	Insufficient or redundant rate (%)	2.13	2.13	2.08	−16.50	−22.22	0.00	−25.81
Yueyang	Actual value	20,694.00	1,003,192.00	91.22	2058.00	1151.00	96,494.00	1759.00
	Projection value	21,059.18	1,207,652.05	92.83	1846.92	1151.00	96,494.00	1464.42
	Insufficient or redundancy value	365.18	204,460.05	1.61	− 211.08	0.00	0.00	− 294.58
	Insufficient or redundant rate (%)	1.76	20.38	1.76	−10.26	0.00	0.00	−16.75
Yongzhou	Actual value	14,395.00	780,164.00	91.94	1343.00	831.00	88,198.56	1043.00
	Projection value	14,536.86	806,190.70	92.93	1333.10	831.00	70,611.25	1043.00
	Insufficient or redundancy value	141.86	26,026.70	0.99	−9.9	0.00	−17,587.31	0.00
	Insufficient or redundant rate (%)	0.99	3.34	1.1	−0.74	0.00	−19.94	0.00
Yiyang	Actual value	17,231.00	714,132.00	90.73	1537.00	866.00	71,227.00	1474.00
	Projection value	17,292.63	822,524.68	91.06	1422.99	866.00	71,227.00	1076.99
	Insufficient or redundancy value	61.63	108,392.68	0.33	−114.01	0.00	0.00	− 397.01
	Insufficient or redundant rate (%)	0.36	15.18	0.36	−7.42	0.00	0.00	−26.93

Positive number represents insufficient rate, negative number represents redundancy rate

*Y*_*1*_ Number of live births; *Y*_*2*_ Number of outpatients and emergency visits; *Y*_*3*_ System management rate for children under 3 years old

*X*_*1*_ Number of health personnel; *X*_*2*_ Number of beds; *X*_*3*_ Operating areas; *X*_*4*_ Number of medical equipment (≥ CNY 10,000)

## Discussion

The evaluation of equity in the allocation of MCH resources in Hunan showed that the Gini values by population were all lower than those by geographic area, suggesting a larger disparity exists in the geographic allocation of MCH resources than in the demographic allocation. The same result has been found in many previous studies [7, 10, 21]. One possible explanation for this finding is that government take the number of health resources per thousand persons, instead of per 10,000 km^2^, as the priority for health resources allocation in China [9, 12] . It may have also contributed to the geographic disparities in the accessibility of health-care services [26], which is incompatible with the goal of “Universal Health Coverage” advocated by WHO that recommends every people could get access to affordable and quality healthcare services [27]. The government should focus more on the inequity of resources allocation in terms of geographical dimension when making the health planning, and improve the accessibility of MCH for people living in the remote and economically underdeveloped regions.

The Gini values for the number of medical equipment (≥ CNY 10,000) and midwives by geographic area exceeded 0.4, indicating the distribution of these two resources were inequitable among different regions. The medical equipment (≥ CNY 10,000) is representative a higher-quality health service that plays a critical role in patient’s diagnosis and treatment, such as the positron emission computed tomography, gamma knife and linear accelerators [28]. While a proportion of people may need to travel long distances in order to access necessary examination and treatment due to the inequitable allocation of MCH resources by geographic area, and this will therefore affect the improvement of their health outcomes [11]. Policy makers need to consider the geographical accessibility of health services when planning the number of medical devices at different regions. Midwives are the backbone of MCH system, playing a vital role in providing health services for mothers and children [29]. However, midwives in China even in the world are insufficient in total quantity [30, 31]. Additionally, it is normal that health-workers tend to serve in developed regions with better economic conditions and more opportunities for career development, which may further enlarge the gaps in the number of midwives between different regions. Increasing the number of well-educated and trained midwives will be the key to the achievement of universal health coverage [31].

Less than one-third regions in Hunan achieved high efficiency in the allocation of MCH resources. While more than 40% of regions were found to be relatively inefficient in the allocation of MCH resources with decreased return to scale, suggesting that those inefficient regions were using more health resources than needed to produce the current amount of health services. Results from the input-output projection value analysis showed that the relative inefficient MCH resources allocation are concentrated on the economically underdeveloped regions in Hunan (e.g., Huaihua, Yongzhou) [19]. According to the projection value of input indicators, almost all inefficient regions had input redundancies, especially in the number of health-workers and medical equipment (≥ CNY 10,000). From the projection value of output indicators, the number of outpatients and emergency visits, live births and systematic management rate for children under 3 years old still had a lot of room for improvement, particularly the number of outpatients and emergency visits.

There are several possible explanations for this result. One possibility is that the demand of health care services for mothers and children had been overestimated, leading to the input redundancy of MCH resources in these inefficient regions. This possibility may be very small but could not be neglected in the decision-making process. The health institutions in China had been granted autonomy with extremely few government subsidies, during the 1970s- early 2000s market-oriented health reform period [32, 33]. Affected by market competition, they vastly expanded investment, including operating areas extension, beds installation, and the purchase of medical equipment [6]. A previous survey reported that the proportion of using large-scale medical devices was less than 50% in many provinces in China [6]. The under-utilized invested hardware facilities may cause considerable health resources to be wasted, thereby decrease the overall efficiency of resources allocation. This suggests that the potential redundancy of MCH resources should be noticed when health administrators make regional health plans.

Another possibility is that many families prefer to attend MCHHs in well-developed areas, rather than the local MCHHs, to seek higher-quality MCH services, such as tertiary MCHHs in Changsha city in Hunan. Because MCHHs in economically developed areas have more qualified personnel and technical advantages [34]. This phenomenon, in some extent, is suggestive of the regional inequity of the distribution of MCH resources and health utilization, that well-developed regions have more experienced experts and advanced equipments than that in underdevelopment regions. An approach may be helpful to improve the quality of health care services in MCHHs in underdeveloped regions, by sharing training activities in developed regions [35]. It may be useful to improve the quality of medical care in the economic backward areas in a sustainable manner, that medical personnel return to work after a period of receiving the technical training from tertiary hospitals in developed regions.

For the inefficient regions, transferring their excessive input resources to more available health services for mothers and children is another reasonable method to improve the efficiency of MCH resources allocation. The gaps in the performance of MCHHs between different regions may be another factor leading to the fact that the amount of services produced by MCHHs in these inefficient regions, given the same level of inputs, was relatively smaller, compared to the counterparts in efficient regions. In this regard, focus should be on strengthening capacity for increased services delivery. The MCHHs in inefficient regions are primarily tasked with improving staff performance and the quality of their management practices in order to effectively utilize inputs and provide available health care services for the mothers and children in economically backward areas [36].

In fact, the real reasons for the inefficiency of MCH resources allocation are complex. Multiple factors such as the demand of health services, geographical distribution, quality of medical care, hospital management and patient preference should be considered to optimize the allocation of health resources. Further investigations on the factors that will influence the efficiency of MCH resources allocation is needed before government make a decision on whether decrease health resources or increase health services to improve efficiency of the MCH resources allocation.

There are several limitations in this study. First, we could not observe the changes in equity and efficiency of MCH resources allocation in Hunan over time because only 1-year data was used in this study. Longitudinal data should be collected in future studies to provide more valuable information for policy makers to improve the health status for mothers and children. Second, although the selection of indicators is based on the published literature review and expert opinions, other indicators associated with health resources allocation, such as the number of discharged patients and systematic management coverage for pregnant women, were not investigated in this study. Future studies should consider additional indicators to have a comprehensive evaluation of the MCH resources allocation. Third, when estimating the efficiency of MCH resources allocation at a regional level, the sum of inputs/outputs of all MCHHs in each region was used to estimate the efficiency scores of each region (DMU), except for the indicator of system management rate for children under 3 years old. An averaged value was regarded as the system management rate of each region. Although this data processing method has been commonly used in previous studies [9, 11, 12], this estimated result may merely reflect the overall health resources allocation at a region level while may be inconsistent with the real situation of each hospitals. The efficiency evaluation of each MCHH need to be further studied.

## Conclusions

The equity of MCH resources by population size is superior by geographic area and the disproportionate distribution of the number of medical equipment (≥ CNY 10,000) and midwives between different regions was the main source of inequity. Policy-makers need to consider the geographical accessibility of health resources among different regions to ensure people in different regions could get access to available health services. More than 40% of regions in Hunan were found to be inefficient with decreased return to scale in the allocation of MCH resources, suggesting that those inefficient regions were using more health resources than needed to produce the current amount of health services. Further investigations on factors affecting the efficiency of MCH resources allocation is still needed to guide regional health plans-making and resource allocation.

## Supplementary information

**Additional file 1.** The distribution of maternal and child health care resources in Hunan in 2017. Table. The general information on maternal and child health care resources distribution in Hunan in 2017.

## Data Availability

The datasets used and analysed during the current study are available from the corresponding author on reasonable request.

## Abbreviations

MCH: Maternal and child health;
DEA: Data envelopment analysis;
SDGs: Sustainable Development Goals;
WHO: World Health Organization;
GDP: Gross Domestic Product;
MCHHs: Maternal and child health hospitals;
DMU: Decision-making unit;
CCR model: The Charnes, Cooper, and Rhodes model;
BCC model: The Banker, Charnes, and Cooper model;
DRS: Decreasing return to scale
